# The Impact of Mathematical Proficiency on the Number-Space Association

**DOI:** 10.1371/journal.pone.0085048

**Published:** 2014-01-08

**Authors:** Danielle Hoffmann, Christophe Mussolin, Romain Martin, Christine Schiltz

**Affiliations:** 1 University of Luxembourg, Walferdange, Luxembourg; 2 Université Libre de Bruxelles, Brussels, Belgium; University of Muenster, Germany

## Abstract

A specific instance of the association between numerical and spatial representations is the SNARC (Spatial Numerical Association of Response Codes) effect. The SNARC effect describes the finding that during binary classification of numbers participants are faster to respond to small/large numbers with the left/right hand respectively. Even though it has been frequently replicated, important inter-individual variability has also been reported. Mathematical proficiency is an obvious candidate source for inter-individual variability in numerical judgments, but studies investigating its influence on the SNARC effect remain scarce. The present experiment included a total of 95 University students, divided into three groups differing significantly in their mathematical proficiency levels. Using group analyses, it appeared that the three groups differed significantly in the strength of their number-space associations in a parity judgment task. This result was further confirmed on an individual level, with higher levels in arithmetic leading to relatively weaker SNARC effects. To explain this negative relationship we propose accounts based on differences in access to qualitatively different numerical representations and also consider more domain general factors, with a focus on inhibition capacities.

## Introduction

The way humans represent numbers has been a recurrent subject of interest both in science and education. Evidence for a close connection between numerical and spatial representations dates back to the 19^th^ century, when Galton described how people visualised numbers and number ranges, which took sometimes very elaborate spatial forms [1]. Since these early observations, there has been extensive behavioural evidence for the relation between numbers and space (for reviews, see [2]–[4]).

One classical demonstration of the number-space association is the so-called SNARC (Spatial Numerical Association of Response Codes) effect. Dehaene and colleagues [5], [6] described that, during a binary classification task on single Arabic digits, adult participants were systematically faster to respond to small numbers with their left hand and to large numbers with their right hand. Classically, the SNARC effect is thought to reveal a spatial code in which numbers are represented horizontally [7] and from the left to the right (in Western participants) [6] according to their magnitude (mental number line hypothesis). This code is supposed to be activated automatically each time Arabic digits are processed - even in classification tasks that do not require access to number magnitude, such as in parity judgment tasks [6]. More recently, an alternative dual processing model has been proposed by Gevers and colleagues [8]–[10]. According to this account numerical magnitudes are associated with a verbal-spatial code ([11]–[13], see also [14]) such that the verbal concepts of “small”/“large” are associated with the concepts of “left”/“right” respectively [11], [15]. Whereas the mental number line theory proposes that number-space interactions affect semantic representations, the latter model situates the origin of the SNARC effect at response selection stages [9], [16], [17]. A third framework, elaborated by Fias and colleagues, challenges the role that cardinal long-term memories are thought to play in number-space associations [18], [19]. This working memory (WM) account postulates that the SNARC effect depends on serial position in WM [18], [19], such that numbers (or any items) forming the beginning/end of a WM sequence are associated with the left/right side of space, respectively ([19], see also [20]).

Despite this rich theoretical context and although it has often been replicated (e.g. [21], [22], see also [23], [24]), the SNARC effect is also characterized by high inter-subject variability that is still poorly understood. According to Wood, Nuerk, & Willmes [25], [26], the proportion of participants showing a SNARC effect varies between 65% and 75%. Individual variability with respect to SNARC effects has been attributed to relatively unspecified sources such as “individual differences in implicit mental representation of numbers, which differs from left to right representation” [27], but the more concrete roles of finger counting habits [28]–[30], response speed [10], [24], [31] and gender [32] have also been described. Inconsistent results in number-space associations appear to be a signature of all tasks assessing the interaction of numerical and spatial representations. The finding that numbers orient visuo-spatial attention according to their magnitude (small numbers to the left, large numbers to the right) (e.g. [33]) is for instance known to be highly vulnerable to task manipulations and context [34]–[36] and could not be replicated in all studies [37]. Consequently, in order to better understand the nature of spatio-numerical associations it will be necessary to further investigate which factors influence them and how they lead to individual differences in number-space interactions.

When attempting to explain the individual variance observed in SNARC tasks, it seems particularly interesting to consider the role of mathematical proficiency. In SNARC experiments, participants are indeed required to perform basic numerical tasks, such as parity or magnitude judgments. Data from the individual differences as well as the neuropsychological literature both in children and adults demonstrate that performance in basic number tasks is systematically related to participants’ math proficiency. Recent studies exploring individual differences in number approximation tasks highlight the relationship between mathematical abilities and approximate number sense (for adults see [38]–[41]; for children see [42]–[45]). In children, further evidence comes from number line estimation tasks [46]–[49] and number comparison tasks [50]–[52]. Accordingly, the strength of number-space interactions (i.e. SNARC effects) associated with these basic number tasks might also be affected by individual differences related to math expertise.

Additionally, the neuropsychological literature repeatedly demonstrated that children and adults with developmental dyscalculia differ from their normally achieving peers in basic numerical processing tasks [53]–[58]. These differences are thought to arise either from differences in number magnitude representations [55], [59]–[61], in accessing number magnitude representations [58], [62]–[64] or in abilities depending on more domain general factors such as working memory or inhibition [65]–[71].

Given these results we hypothesized that the access of numerical representations and/or domain general factors such as working memory might modulate the way numerical and spatial information is integrated, leading to differential SNARC effects according to math proficiency. Young adults with high proficiency levels in mathematics might for instance display weaker SNARC effects because they have a better access to numerical representations allowing them to retrieve parity status information more easily. This would in turn decrease the interference of task irrelevant factors, such as digit magnitude in parity judgment SNARC tasks (see also [72]). Concretely, parity status for one-digit Arabic numbers is thought to be retrieved from associative semantic networks [67], [73] along with other arithmetic properties [6] via a spreading activation process [65], [74]. Access to number representations has been shown to be modulated by math proficiency [58], [62]–[64], [75], [76], with lower math proficiency leading to less automatic access to number representations. Less automatic access, on the other hand, is known to result in more executive load [77], which in turn has been shown to prevent prioritization of target processing as well as inhibition of distractor processing [78]. From this point of view, math proficiency might well contribute to inter-individual variability in parity judgment SNARC tasks.

Numerical proficiency was already evoked in previous discussions of individual variability in SNARC tasks (e.g. [6], [31], [79]), but comprehensive studies remain scarce. Former studies exploring the influence of math proficiency and math training on the quality of participant’s SNARC effect revealed tendencies towards a modulatory impact; however, they lacked statistical power to draw firm conclusions concerning their influence [6], [79]. So far only one study made a consequent effort to collect data in a large population (n =  71) of university students from study fields with two different math-level requirements [31]. Contrary to their expectations the authors could, however, not observe any systematic relation between math proficiency and the strength of SNARC effects, calling for more work on this topic.

The present study aimed to further investigate the influence of mathematical proficiency on number-space interactions as indexed by the SNARC effect, controlling for more domain general factors such as processing speed and visuo-spatial working memory. In order to explore a large range of mathematical proficiency levels, we formed three different math proficiency groups by recruiting university students from study fields with math-heavy vs. math-light curricula, including also students reporting specific math difficulties (i.e. Math Expert group, Control group, Math Difficulties group). To prevent potentially confounding gender-effects the groups were balanced for gender as far as possible, leading to gender-matched groups of problem-free participants. To complement the math proficiency characteristics indicated by the study field background, we also assessed participants’ arithmetic skill level with respect to the basic arithmetic operations. Using a parity judgment task, we expected that participants with more efficient numerical processing skills would display weaker SNARC effects. Concretely, we hypothesized that during a parity judgment, task-irrelevant magnitude information - and the associated spatial code leading to SNARC effects - would interfere less in adults that are highly trained and proficient with numbers. In contrast, adults that are weaker in mathematics or report specific difficulties in this domain should reveal stronger SNARC effects, because the irrelevant magnitude information (and the associated spatial representations) interferes more strongly with the parity judgment task. Moreover, if the impact of mathematical proficiency levels on the individual differences in SNARC effect strength is specific, we further expected that more general factors such as processing speed and working memory cannot explain the relationship between math proficiency and SNARC effect strength.

## Methods

### Ethics statement

In accordance with the National Ethics Committee (CNER) approving the present study, all participants gave written informed consent prior to participating.

### 1. Participants

A total of 95 university students took part in the present study in exchange for payment. In order to control for equal gender and math proficiency level distribution, the students were recruited on behalf of their study fields. Only students of curricula with either a clear predominance (Math Expert group) or absence of explicit daily number and mathematics use were included in the study. Within the latter group we further distinguished students reporting average math abilities (Control group) from those reporting specific difficulties with mathematics (Math Difficulties group).

### 1.1. Math Expert group

The Math Expert group (ME) included 38 students with a study field having a strong numerical demand (e.g. mathematics, engineering and sciences); 19 were female, 5 were left-handed and their mean age was 23.2 years (SD = 2.5, range  = 19–29 years). None of the participants reported specific difficulties with mathematics and/or had a diagnosis of a learning disability.

### 1.2. Control group

The Control group (CON) included 38 university students enrolled in a field of study with no explicit use of mathematics (e.g. literature, linguistics and law), 20 participants were female, 1 was left-handed, and their mean age was 23.1 years (SD = 3.1, range  =  18–33 years). None of the participants reported specific difficulties with mathematics and/or had a diagnosis of a learning disability.

### 1.3. Math Difficulties group

The Math Difficulties group (MD) included 19 university students enrolled in a field of study with no explicit use of mathematics (e.g. literature, linguistics and law) and who reported experiencing specific difficulties with numbers. Seventeen were female, 1 was left-handed and their mean age was 24.8 years (SD = 3.8, range  =  20–31 years). None of these participants had been diagnosed with a learning disability, but all reported having difficulties with numbers specifically.

### 2. Materials & Procedure

The computerized tasks were programmed in E-prime (Version 2.0.8.79; [80]) and administered using a Lenovo ThinkPad 61 Tablet Laptop with a 12.1 in. color monitor (1024×768 Pixels), in a quiet room.

### 2.1. Arithmetic, working memory and processing speed assessment

**Arithmetic competency.** To assess individual arithmetic proficiency, both timed and untimed arithmetic tests were administered to the participants.

**Untimed arithmetic paper-pencil test: “Arith”.** We used the battery of arithmetic operations developed by Shalev et al. [81] and adapted by Rubinsten & Henik [58]. This battery assesses proficiency in arithmetical operations including addition, subtraction, multiplication and division problems, ranging from number facts (5 problems/operation) over complex arithmetic (8 problems/operation) and decimals (4 addition and 4 subtraction problems) to fractions (5 problems/operation). Instead of errors (cf. [58]) we scored 1 point per correct problem, hence participants could reach a maximum score of 80 points.

**Timed computerized arithmetic task: “FastMath”.** The timed computer-based calculation task was developed and described in detail by Mussolin and colleagues [56]. The participants were asked to solve addition, multiplication and subtraction problems on one- or two-digit Arabic numbers. During each trial an arithmetic problem was presented centrally in Times New Roman font, pt. size 50, along with two possible response propositions presented below. Participants had to press the key (“A” or “L” on a standard QWERTZ keyboard) on the side corresponding to the correct response. The experiment consisted of three blocks of 20 problems, one per arithmetic operation. Order of stimuli presentation and position of the correct response were randomized across trials.

**Visuo-spatial working memory (VSWM).** This paper-pencil test is based on the Visual Pattern Test [82], [83] and provides a measure of the spatial-simultaneous working memory span. Participants were presented a series of matrices, progressively increasing in size, where half of the cells were filled in black. After the presentation phase, the participants had to reproduce the memorized patterns of filled squares in a blank matrix. The highest number of correctly recalled filled squares was taken as measure of VSWM span.

**Processing speed (GPS).** Both general and numerical processing speed measures were obtained in all participants.

**General processing speed (GPS).** To assess GPS, participants performed a speeded matching to sample task (see also [56]). In each trial, a shape was presented centrally on the screen and just below the same shape was displayed with a new shape. Participants simply had to press the key on the side corresponding to the matching shape. Twenty trials of this task were performed at the end of the timed computerized arithmetic task.

**Parity judgment reaction times (PJ-RT).** The SNARC effect was evaluated using a parity judgment task. During this task participants’ response times (and accuracy scores) are recorded in order to compute the SNARC effect (for details cf. 2.4). However, the response times collected in this task can also be used to assess participants’ processing speed for this specific numerical task. This information concerning the response times during parity judgment (PJ-RT) complements the above-mentioned indication on participants’ general processing speed in a non-numerical task.

### 2.2 Descriptive information on the group composition

Details of the descriptive information concerning the three populations (number of men and women, mean age, number of left-handers) are given in Table 1. The mean ages did not differ significantly across the three groups (*F*(2,92)  = 2.4, *p*>0.05), nor did the number of left-handers (*χ^2^*(2)  = 3.2, *p*>0.05). The first two groups matched closely for gender (*χ^2^*(1)  = 0.05, *p*>0.05), but due to the composition of the third group (MD) overall the number of men/women differed significantly between the three groups (*χ^2^*(2)  = 9.2, *p*<0.05).

**Table 1:** Descriptive information and mean performance for the three groups in the general assessment tasks.

	ME	CON	MD	*F*
	Mean *(SD)*	Mean *(SD)*	Mean *(SD)*	
Descriptive Information				
*N*	38	38	19	
Gender (M/F)	19/19	18/20	2/17	χ^2^ (2) = 9.2^*^
Age (in years)	23.2 (2.5)	23.1 (3.1)	24.8 (3.8)	2.4; η^2^ = 0.05
Handedness (R/L)	33/5	37/1	18/1	χ^2^ (2) = 3.2
*Arithmetic*				
Arith (ACC)	92.4 (6)_a_^***^	82.8 (13)	72.7 (19)_b_^*^	16.31^***^; η^2^ = 0.27
FastMath (ACC)	94 (3)_a_^†^	92.2 (5.3)	90.5 (5.6)	4.26^*^; η^2^ = 0.08
FastMath (RT)	2688 (891)_a_^*^	3234 (1124)	4850 (2168)_b_^***^	9.91^***^; η^2^ = 0.27
zArithmetic	1.27 (1.2)_a_^**^	–0.14 (2.1)	–2.26 (2.9)_b_^**^	17.09^***^; η^2^ = 0.30
*Response Speed*				
GPS	715 (273)	837 (679)	1086 (792)	2.29; η^2^ = 0.05
PJ-RT	535 (68)_a_^*^	575 (79)	619 (75)_ b_^*^	8.47^***^; η^2^ = 0.16
*Visuo-spatial working*				
Memory span	9 (1.9)_a_^*^	8.1 (1.7)	7.8 (1.7)	3.5^*^; η^2^ = 0.07

*Note.* Standard deviations are shown in parentheses; RTs are given in ms and ACC in percent; significant differences are indicated by ^*^ p<0.05; ^**^ p<0.01; ^***^ p<0.001; († p = 0.06). A significant contrast between ME and CON is indicated by “a” followed by the level of significance; a significant contrast between MD and CON is indicated by “b” followed by the level of significance. Welch’s *F* is indicated in case the homogeneity of variances assumption was violated.

Concerning arithmetic tests, we found a significant group effect for all measures, confirming the distinct mathematical proficiency levels of the three groups. In the untimed Arith battery, planned comparisons indicated that the participants of the CON group made significantly more mistakes than the participants of the ME group (*p*<0.001), but significantly less mistakes than the participants of the MD group (*p*<0.05). Regarding the accuracy in the timed FastMath test, there was a similar trend between the ME and the CON group (*p* = 0.06) but no difference between the CON and the MD group. On the other hand when considering the speed with which the participants solved the arithmetic problems in the FastMath test, the participants of the CON group were significantly slower than the participants of the ME group (*p*<0.05), but significantly faster than the participants of the MD group (*p*<0.01).

In order to have a single arithmetic proficiency measure, we computed the composite score zArithmetic from the normed values of the arithmetic tests available: zArithmetic  =  zArith (ACC) + zFastMath (ACC) – zFastMath (RT). As expected, there was a significant effect of group on this composite score, with the participants of the CON group obtaining a lower value than the participants of the ME group (*p*<0.01) and a higher value than the participants of the MD group (*p*<0.01). These different composite scores reflect the fact that the CON group was slower and more error prone in arithmetic problem solving than the ME group, but faster and more correct than the MD group.

There was no group effect in general processing speed as assessed by the GPS task (*p*>0.05). In contrast, the three groups differed significantly in PJ-RT, with ME participants being significantly faster than CON participants (*p*<0.05) and CON participants significantly faster than MD participants (*p*<0.05) to judge the parity of single digits. The main effect of group was also significant in the VSWM task, because the ME had a larger visual short-term memory span than the CON group (*p*<0.05). The 2 weaker math groups (CON and MD) on the other hand achieved similar results (*p*>0.5). However, all three groups were within one standard deviation of the mean of the normative data of the VSWM task (9.08 ± 2.25, see [82]).

### 2.3. Experimental Task: SNARC (computerized)

In order to assess the participant’s SNARC effect, they were administered a parity judgment task on single digits. The design of this task was adapted from Dehaene et al. [6]. During the parity judgment task, the participants had to judge whether a centrally presented Arabic digit was odd or even. Each trial started with an empty black-bordered transparent square on a white background (sides 100 pixels, border 2 pixels). After 300 msec, one of ten possible stimuli (Arabic digits 0, 1, 2, 3, 4, 5, 6, 7, 8 or 9) presented in black on a white background in font Arial pt. size 48, appeared at the center of the square and remained for 1300 msec. The intertrial interval consisted of a blank screen of 1300 msec. The stimuli were presented in a pseudo-random order, no number appeared twice in a row, and the correct response could not be on the same side more than three times consecutively. Responses were given by pressing the “A” or the “L” key of a standard QWERTZ keyboard.

Each participant completed two blocks, one in each mapping (in one block “A” was assigned to “odd”, in the other one “A” was assigned to “even”); block order was counterbalanced across participants. Each block started with 12 to 20 training trials, depending on response accuracy. An accuracy threshold of 70% correct answers had to be reached in order to proceed directly after 12 training trials to the experimental trials, if the threshold was not reached, another 8 training trials were administered before the experimental trials started. The experiment itself consisted of 180 trials, 90 trials per block; each number was presented 9 times per block.

Participants all started with the SNARC task, then “FastMath”, followed by “GPS”, the Arith paper-pencil test and then the VSWM task. The participants were part of a larger project including additional behavioral measures, not reported here and administered at the end of the testing for the present study.

### 2.4. Statistical Analyses

Prior to data analyses, error trials (with respect to the parity judgment) were removed from the data (5.78% of all trials). A univariate ANOVA revealed that the three participant groups did not differ in error rates (*F*(2, 92)  = 2.4; *p*>0.05). Reaction times (RTs) longer or shorter than 2.5 standard deviations from the individual mean were considered outliers and removed (2.67% of all trials).

In order to control for possible biases of parity status on lateralized RT (Markedness Association of Response Codes effect-MARC, see [84]), we conducted a repeated measures ANOVA with Parity status (odd, even) and Response side (left, right) as within subjects variables and Group as a between subjects factor. There was no interaction between Parity status and Response side (*F*(1, 92) = .56; *p*>.4; η^2^ = .006) and no interaction of a MARC effect with Group (*F*(2, 92) = .36; *p*>.7; η^2^ = .008), hence we did not further investigate MARC effects.

For almost two decades, studies investigating the SNARC effect used regression analysis methods for repeated measures data following Lorch and Myers [85] as suggested by Fias and colleagues [21]. This method implies calculating mean RTs for each digit and response side (left/right) and for each individual subject separately. Individual RT difference scores (dRT) are then computed by subtracting for each digit the mean RT of left-sided responses from the mean RT of right-sided responses. The resulting dRT scores are submitted to a regression analysis, using the magnitude of individual stimuli numbers as predictor variable. Negative regression weights (slopes) reflect SNARC effects in the expected direction (faster left/right-sided RT for small/large digits respectively).

Recently, the habit of only using regression slopes to determine the strength of the SNARC effect has been questioned [86], [87]. These authors argue that even though the Lorch and Myers regression method allows testing the significance of the linear relation between numbers and dRT (i.e. the expected RT difference between right and left hands for a given change of magnitude), it does not provide an estimate of the correlation between the dRT and number magnitude. Hence it is reasoned that individual slopes should not be interpreted as effect sizes of the SNARC effect [87]. Additionally, Tzelgov and colleagues propose to use magnitude as the predictor variable instead of individual numbers in order to avoid MARC effects (see [84]).

Taking into account these recent methodological criticisms, we will analyze hypothesized group effects in the SNARC effect by conducting a repeated measures ANOVA on dRT with Magnitude as a within subject and Group as a between subjects factor as suggested by Pinhas and Tzelgov and colleagues [86], [87]. In order to avoid bias induced by possible MARC effects, we first collapse RT to an even and an odd digit, resulting in 5 Magnitude categories: Very small (0, 1), Small (2, 3), Intermediate (4, 5), Large (6, 7) and Very large (8, 9) for each subject and response hand separately. We then compute for each subject dRTs for each Magnitude category. In this approach, a SNARC effect is revealed by a significant main effect of Magnitude associated with a significant linear trend. Additionally, this method provides us with an effect size of the linear trend. In the present study, the group factor differentiates between the three experimental groups (ME, CON, MD).

Additionally, we computed regression slopes (SNARC slope) with individual numbers as proposed by Fias and colleagues [21] since they a) directly reflect the interaction between numerical magnitude and response side b) highlight the inclination and direction of lateralized RT effects associated with the underlying hypothetical number line and c) permit direct comparison with the slope results reported in previous studies on the SNARC effect over the last 17 years. To complete our analyses, we report individual effect size measures of the number-space interaction, which comprise information on the scattering of the data points around the linear regression slope. Thus, we computed individual correlation analyses between dRT and Magnitude, yielding individual Pearson’s *r*, which were then transformed to z-scores using a Fisher transformation in order to have individual (and normally distributed) measures that could be correlated with the other variables (e.g. arithmetic scores).

According to our hypotheses, the ME group, which is expected to have the weakest SNARC effect, should have the least negative SNARC slope compared with the other two groups, whereas the MD group should have the most negative SNARC slope, reflecting the most pronounced number-space interaction. Similar results are also expected when computing the relation between individual slopes and arithmetic abilities. In contrast, we did not have any specific hypotheses regarding the impact of arithmetical proficiency on individual SNARC effect sizes since they rather reflect the scattering of data points around the linear regression than the shape (inclination and direction) of the number-space interaction itself.

## Results

### 1. The SNARC effect: group contrast analysis

**Figure 1:**
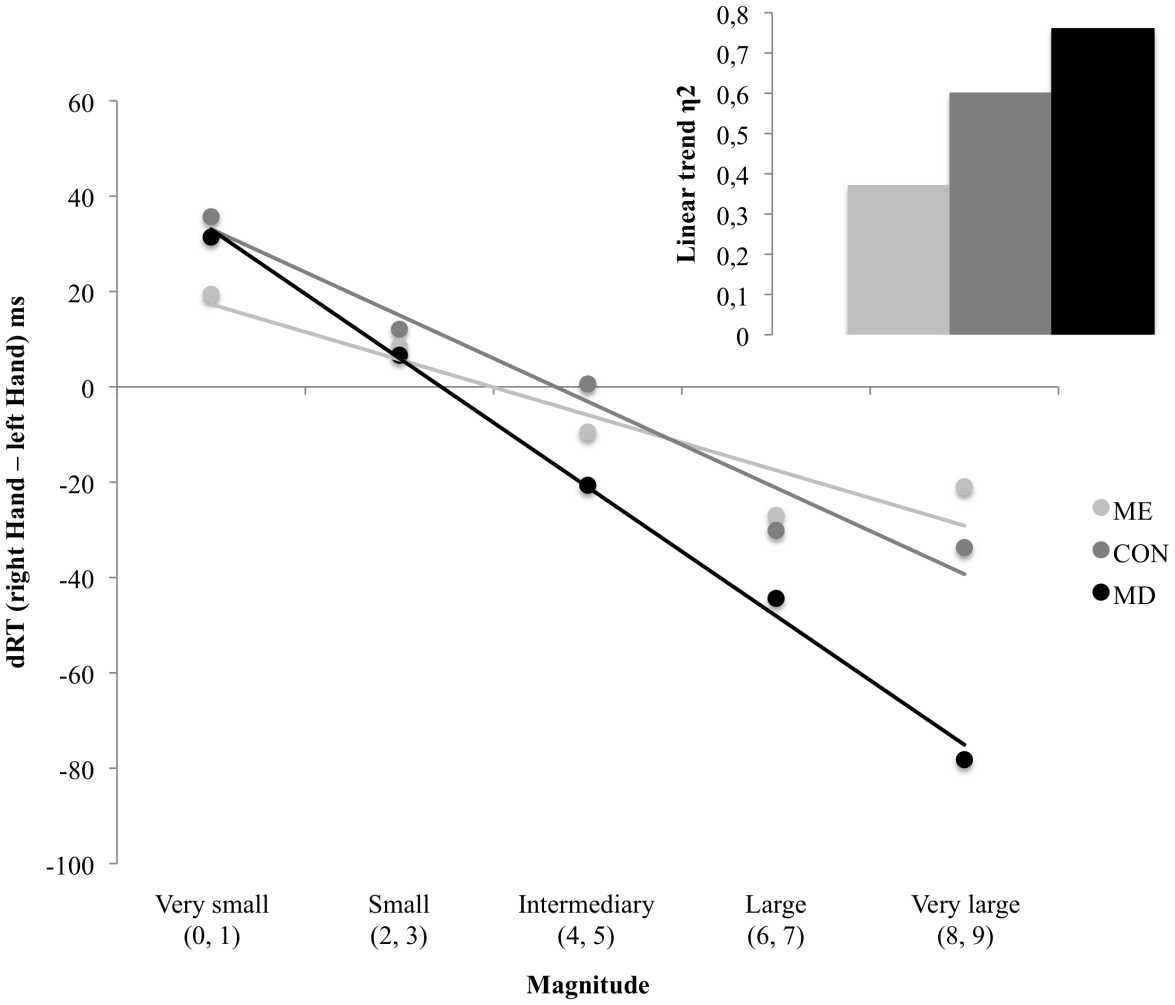
dRT (in ms) as a function of Magnitude category by group. Lines represent the linear fits on group data. A negative relation indicates the presence of a SNARC effect. The inset depicts linear trend effect sizes per group.

Following the approach suggested by Pinhas, Tzelgov and colleagues [86], [87], we conducted a repeated measures ANOVA on dRT with Magnitude (Very small, Small, Intermediate, Large and Very Large) as a within subjects variable and Group as a between subjects variable. This analysis revealed a significant main effect of Magnitude (*F*(4, 368) = 39.9; *p*<0.001; η^2^ = 0.303) associated with a significant linear trend (*F*(1, 92) = 129.8; *p*<0.001; η^2^ = 0.59), meaning that there was a significant SNARC effect in the entire sample. An interaction between Magnitude and Group confirms our hypothesis that the SNARC effect differed between the experimental groups (*F*(8, 368) = 2.36; *p*<0.05; η^2^ = 0.05). Evaluating the math proficiency groups separately, the analysis reveals a significant SNARC effect in every group (ME: main effect of Magnitude *F*(4, 148) = 7.99; *p*<0.001; η^2^ = 0.18; associated linear trend *F*(1, 37) = 21.2; *p*<0.001; η^2^ = 0.37; CON: main effect of Magnitude *F*(4, 148) = 16.37; *p*<0.001; η^2^ = 0.31; associated linear trend *F*(1, 37) = 54.9; *p*<0.001; η^2^ = 0.60; MD: main effect of Magnitude *F*(4, 72) = 16.8; *p*<0.001; η^2^ = 0.48; associated linear trend *F*(1, 18) = 57.3; *p*<0.001; η^2^ = 0.76; see also Figure 1). *(These results remained the same when RT for « 0 » and « 5 » were excluded from the analyses (F(6, 276) = 3.43; p<0.01; η^2^ = 0.07; see* *[84]* *,* *[88]* *for the special status of « 0 »; however, these results need to be interpreted with caution due to the reduced set-size on which they are computed. Additionally, they are based on a post-hoc simulation not reflecting actual experimental settings known to critically influence SNARC effects, i.e.* *[6]* *.)*

As mentioned in the methods section, in addition to the group analyses, we computed individual SNARC effect measures. Accordingly, we analyzed our results following Fias and colleagues [21], obtaining regression slopes in order to allow comparison with SNARC studies published previously. We also computed individual effect size measures as described in the methods section. The regression analyses of individual digits on dRT revealed a significant negative (unstandardized) slope in the ME group (B = –5.25, one-tailed comparison of B to zero: *t*(37) = 3.92; *p*<0.001, effect size: –0.46), in the CON group (B = –8.82, one-tailed comparison of B to zero: *t*(37) = 7.15; *p*<0.001, effect size: –0.77) and in the MD group (B = –13.23, one-tailed comparison of B to zero: *t*(18) = 9.38; *p*<0.001, effect size: –1.11) respectively.

A one-way ANOVA on SNARC slopes and SNARC effect sizes with group as a between-subjects factor revealed that the groups differed significantly with respect to the SNARC effect (slopes: *F*(2,92)  = 7.12, *p*<0.001; η^2^ = 0.13; effect sizes: *F*(2,92)  = 4.01, *p*<0.05; η^2^ = 0.08). There was a significant linear trend, (slopes: *F*(1,92)  = 14.2, *p*<0.001, η^2^ = 0.13; effect sizes: *F*(1,92)  = 8.01, *p*<0.01; η^2^ = 0.08), indicating that the strength of the SNARC effect increased from ME over CON to MD. In other words, the SNARC increased with decreasing math proficiency of the groups. Planned contrasts revealed that the ME group had a significantly weaker SNARC effect than the CON group (slopes: *t*(92)  = 2.04, *p*<0.05 (one-tailed), *r* = 0.21; effect sizes: *t*(92)  = 1.64, *p* = 0.05 (one-tailed), *r* = 0.17), and the CON group had a weaker SNARC effect than the MD group, (slopes: *t*(92)  = 2.07, *p*<0.05 (one-tailed), *r* = 0.21; effect sizes: *t*(92)  = 1.44, *p* = 0.08 (one-tailed), *r* = 0.15). Reflecting the group differences in SNARC effect sizes, 74% of the participants in the ME group had a negative SNARC slope, 89% of the participants in CON group, and 100% of the participants in the MD group.

Our findings were confirmed by an ANCOVA including GPS, PJ-RT and VSWM span as covariates, yielding a main effect of group on SNARC slopes (*F*(2,89)  = 3.7, *p*<0.05; η^2^ = 0.08) and effect sizes (*F*(2,89) = 3.3, *p*<0.05; η^2^ = 0.07), but no effect of either GPS, PJ-RT or VSWM (all *p*s>0.14). These analyses confirm that the strength of spatio-numerical interactions was significantly modulated by mathematical proficiency groups, even when controlled for potential confounds due to differences in processing speed (GPS, PJ-RT) or visuo-spatial working memory (VSWM span).

### 2. The SNARC effect: individual correlation analysis

To investigate the SNARC effect on an individual level, Pearson correlation analyses were conducted (see Table 2). The correlation analyses revealed that GPS and PJ-RT were related to each other and both correlated negatively with the zArithmetic score. Hence, participants that were faster in the speeded matching to sample task were also faster to do parity judgments. Moreover, they performed better in the arithmetic tests. In contrast to GPS, PJ-RT was additionally related to VSWM, participants that were faster in doing parity judgments had a better VSWM span. VSWM span was also related to the zArithmetic score, revealing that participants with a larger VSWM span also did better in the arithmetic tests.

**Table 2:** Correlations between different variables (N = 95).

	1.	2.	3.	4.	5.
1. SNARC slope					
2. SNARC effect size	.68^**^				
3. zArithmetic	.28^**^	.17^♯^			
4. VSWM	.12	.15	.39^**^		
5. GPS	–.19	–.04	–.27^**^	–.17	
6. PJ-RT	–.30^**^	–.08	–.37^**^	–.31^**^	.38^**^

*Note.* ^#^ *p*<0.1; ** p<0.01.

The SNARC effect measures of slope and effect size were related to each other; the steeper the participant’s slope, the more important his or her effect size. Furthermore, SNARC slopes correlated positively with the zArithmetic score. *(The relation between the SNARC slope and zArithmetic remained similar when RT for the stimuli « 0 » and « 5 » were not included in the analyses: SNARC slope : r = .19 ; p<.07 ; SNARC effect size : r = .11 ; p>.1.)* This finding illustrates that participants scoring lower in the arithmetic measures also had more negative slopes, meaning more pronounced SNARC effects. SNARC effect sizes and zArithmetic scores were marginally related as well, participants scoring better in arithmetic displayed slightly more important effect sizes. In contrast, participants who scored higher in the arithmetic tests had relatively weaker SNARC effects (i.e. less negative slopes). Moreover, the SNARC slope was related to the speed with which participants performed parity judgments (i.e. PJ-RT). Hence, the slower the participants were to decide whether a digit was odd or even, the steeper their slope. Interestingly, PJ-RT did not correlate with the SNARC effect size. Additionally, participant’s SNARC effects related neither to GPS, nor to their VSWM span; confirming the results obtained in the ANCOVAs of the group analysis.

In order to confirm that the present findings were not exclusively driven by the population reporting specific difficulties with numbers (MD group), we conducted additional correlation analyses excluding this group. A total of 76 participants (ME and CON group members) were included in the analyses, of which roughly half (N = 39) were female. Pearson’s correlation analyses confirmed the previous findings by showing that SNARC slopes were positively related to arithmetic proficiency (*r*  = .25, *p*<0.05) but neither to VSWM span (*r*  = .06, *p*>.5) nor to general processing speed (*r* = –.15, *p*>.1). In contrast to the SNARC slope the relation between SNARC effect size and arithmetic proficiency scores did not reach significance (*r*  = .13, *p*>.1). *(When RTs to the stimuli « 0 » and « 5 » were dropped from the analyses on the reduced sample size correlation coefficients were: slope : r = .16, p>.1 ; effect size : r = .08, p>.4)*

To fully understand the significance of the two SNARC-related measures reported in the present study (i.e. slope and effect size), we investigated the individual relations of each SNARC measure to the variables of interest when the respective other SNARC measure was held constant. Consequently, we conducted two partial correlation analyses. In the first analysis, we investigated the relationship between SNARC slope, PJ-RT and zArithmetic when controlling for SNARC effect-size, whereas in the second analysis we investigated the relationships between SNARC effect size, PJ-RT and zArithmetic when controlling for the SNARC slope measure (see Table 3).

**Table 3:** Partial correlation analyses controlling for SNARC effect size (A) or SNARC slope (B).

(A)		1.	2.	(B)		1.	2.
Effect size	1.zArithmetic			Slope	1.zArithmetic		
	2.PJ-RT	–.37^**^			2.PJ-RT	–.32^**^	
	3.Slope	.22^*^	–.34^**^		3.Effect size	–.02	.19^#^

*Note.* ^#^ *p*<0.1; * *p*<0.05; ** p<0.01.

The partial correlation analyses showed that whereas the relation between zArithmetic and SNARC slope remained significant when controlling for effect size, the marginal relation between zArithmetic and SNARC effect size disappeared when controlling for SNARC slope. Additionally, whereas the previously reported relation between SNARC slope and PJ-RT remained when controlling for effect size, the relation between SNARC effect size and PJ-RT reversed when controlling for SNARC slope.

### 3. The SNARC effect: multiple regression analyses

Finally, we conducted multiple regression analyses in order to investigate relations between the SNARC effect (slope and effect size) and each predictor variable when the effects of the other predictors are held constant. Specifically, we were interested to see whether arithmetic proficiency explained variance of the SNARC effect when GPS, PJ-RT and VSWM capacities were statistically controlled for. Consequently, the following predictors were entered: GPS, PJ-RT, VSWM and zArithmetic. The results show that zArithmetic and PJ-RT were the only predictors that explained a marginally significant amount of variance of the slope of the SNARC effect (see Table 4).

**Table 4:** Results of the regression analysis with SNARC slope as dependent variable.

	B	SE	β	t	p
(Constant)	5.84	8.14		.72	.48
GPS	−.001	.001	−.06	−.52	.61
PJ-RT	−.02	.01	−.22	−1.89	.06
VSWM	−.12	.48	−.03	−.25	.80
zArithmetic	.66	.39	.19	1.70	.09

*Note*. *R* ^2^  = .13; adj. *R* ^2^  = .09; F(4,90) = 3.21, p<0.05.

Considering SNARC effect size, the regression model failed to reach significance (*F*(4, 90) = 0.88; *p*>.1).

Together, the results of the regression analyses confirm the importance of zArithmetic in the observed variability of the SNARC slope. Furthermore, they confirm the importance of PJ-RT in the observed variability of the slope of the SNARC effect reported by previous studies (i.e. [31]). *(Note that the regression models did not reach significance when RT data to the stimuli « 0 » and « 5 » are dropped from the analyses.)*

## Discussion

The present study aimed to investigate whether mathematical proficiency levels affect the strength of number-space interactions as indexed by the SNARC effect. We recruited three groups of university students differing starkly in their mathematical level. Analysis of their arithmetical performance confirmed that students from mathematical study orientations (ME) were more proficient in arithmetic than their study colleagues from non-mathematical orientations. Moreover, within the latter student population those reporting specific difficulties in mathematics (MD) were even less proficient than their colleagues who did not relate specific math problems (CON). Confirming our hypothesis, we observed a main effect of group on the SNARC effect, revealing significantly different number-space interactions in the three groups. Indeed, the CON group displayed a weaker SNARC effect than the MD group, but a stronger SNARC effect than the ME group. Critically, when controlling for general processing speed, parity judgment reaction time or visuo-spatial working memory, the effect of group on the SNARC effect remained. Correlation analyses pertaining to individual performance levels confirmed the group findings and revealed a significant relation between the slope of the SNARC effect and arithmetic scores. Participants scoring lowest in the arithmetic tests displayed the most important SNARC effects (i.e. most negative slopes) and vice versa for participants scoring highest in arithmetic. In line with previous findings, SNARC slopes (but not effect sizes) also related to response times in parity judgment [10], [31]. In contrast, there was no relation between the strength of the SNARC effect and general processing speed or visuo-spatial working memory span, excluding these general accounts for the systematic relationship observed between number-space interactions and mathematical skill level.

The present findings confirm the first indications in the literature [6], [79] that math proficiency modulates the strength of the SNARC effect. However, they contrast with the recent findings of Cipora and Nuerk [31], who failed to find systematic relations between math proficiency and SNARC slopes. As mentioned by Cipora and Nuerk [31], there are a few differences between their study and ours, such as the inclusion of the Arabic digit “0” in our study and different school and language contexts [89], [90]. Cipora and Nuerk [31] also cite the inclusion of a low-skill group as a potentially influencing difference. Our analyses, however, showed that there is a significant group difference when ME and CON are contrasted directly. Moreover the correlation results remain largely unchanged when the MD group is not included. Whereas we balanced the ME and CON groups in number and in gender, this was not the case in the study of Cipora and Nuerk [31]. In their study, only 25% of all participants were in the math group, the other 75% were included in the non-math group. Additionally, their groups were not balanced in gender, with only 28% of female participants in the math group and 83% of female participants in the non-math group. Since there is evidence for stronger SNARC slopes in male participants [32], [51], gender effects might have masked potential math proficiency effects in this population. Indeed, although the impact of math abilities on the SNARC slope was observed robustly and coherently in all analyses of the present study it was only characterized by small to medium effect sizes. Furthermore, both in our and Cipora and Nuerk’s [31] studies the SNARC slope correlated significantly with PJ-RT. When considering that this measure not only reflects participants’ processing speed, but also their numerical ability to judge digit parity, this common finding further supports the existence of a systematic link between mathematical skill level and the SNARC effect.

Finally, it should be mentioned that several other studies which used math abilities as a covariate when investigating SNARC effects also failed to find significant relationships between math proficiency and the strength of numbers-space associations ([32], [91] in adults and [92] in children). However, neither of these studies had made specific efforts to sample participants from a very large range of math proficiency levels. In addition, when math proficiency was assessed the math scores relied on performance in a mixture of arithmetical and other mathematical tasks, precluding a direct comparison with the present approach.

Besides the traditionally reported interaction and slope measures which inform on the presence of significant SNARC effects and characterize their shape, we completed our analyses by indicating also effect size measures of the SNARC effect [86], [87]. In the group analysis the SNARC effect sizes of the three math-proficiency groups decreased linearly with math abilities. This was in line with the observation that a smaller proportion of ME participants (i.e. 74%) had negative SNARC slopes compared to participants of the CON (89%) and MD (100%) groups (hence there was less variance in the presence of SNARC effects in the MD group than in the ME group). In contrast, the correlation and regression analyses indicated that individual arithmetic scores explain considerably less variance of participants’ SNARC effect sizes than of their SNARC slopes. This observation indicates that arithmetic abilities relate to the inclination and direction of the number-space association, rather than the amount of scattering around the linear trend relating lateralized response time to digit magnitude. To explain the negative relationship we expected and observed between the strength of SNARC effects and math proficiency levels, we will discuss distinct (but potentially complementary) hypotheses currently proposed in the literature investigating individual differences in typical and atypical mathematical functioning.

Participants that are more proficient with numbers might have stronger associations between numerical facts than participants with less numerical exposure. In line with data from typical as well as atypical math development indicating that higher math proficiency leads to a ***more automatic access to number representations*** [58], [62−64], [75], , students from the MD group could have less automatic access to numerical representation than those of the CON group, who themselves would access number semantics (i.e. parity status) less easily than their colleagues of the ME group.

Less automatic access to semantic (number) representations results in more executive load [77], which in turn has been shown to prevent prioritization of target processing as well as inhibition of distractor processing [78]. In other words, if access to numerical representations was less automatic in MD or CON group students, they should experience ***higher executive loads when retrieving the parity*** of a given numeral. Higher loads then make it harder to (a) prioritize the parity judgment and (b) inhibit the spatially coded magnitude information that was activated in parallel. As a consequence, the response of mathematically less skilled participants would be more influenced by the task-irrelevant digit magnitude and their SNARC effects would be stronger than those of the ME participants. The proposal that inter-individual differences would be caused by differences in prioritizing parity information and inhibiting irrelevant magnitude information would be in line with the theoretical framework of the dual processing model [8], [9], [12]. This view is supported by behavioral and imaging studies that have found the SNARC effect to be localized at response related stages, as opposed to representational stages [8], [9], [16], [17]. Specifically, using event-related potentials (ERPs) to investigate the functional locus of the SNARC effect during parity judgment, Keus and colleagues [17] only found evidence for the SNARC effect in response-locked ERPS, but not in stimulus-locked ERPs. Furthermore they found evidence that the SNARC effect is localized at response selection stages that take place prior to response preparation and execution stages. Additional support for the dual processing model is provided by the correlations between parity judgment response times and the slope of SNARC effect that were observed here and in previous studies [10], [31]. On the other hand, as in Cipora and Nuerk [31] general processing speed assessed in an independent task did not relate to SNARC effects.

Consistent with the proposal that SNARC effects are localized at response selection stages, another hypothesis implicating ***domain-general factors*** might explain the findings of the present study. Working memory and inhibition deficits have repeatedly been proposed to be related to arithmetic proficiency in healthy adults [65] as well as in participants suffering from DD [66]–[71]. According to these findings, MD and CON participants should in general have a higher sensitivity to activation-based interference [65], [66] and lower capacities to inhibit irrelevant information [70], [71]. Following the above-mentioned principles, these executive difficulties would again lead to less efficient inhibition of task-irrelevant magnitude information (and consequently larger SNARC effects) when mathematically weaker students perform a parity judgment task. A general finding supporting this latter hypothesis is the increase of the SNARC effect with age [24], [93] and declining general inhibition capacities [94]. In this recent study we assessed the influence of cognitive inhibition abilities on the strength of SNARC effects in younger and elderly participants and thus observed a significant correlation between Stroop and SNARC effects. In contrast, the present study indicates that visuo-spatial working memory capacity does not influence the strength of the SNARC effect. Indeed we observed a significant difference between the VSWM spans of the math groups. But despite this generic group effect, visuo-spatial working memory span did not correlate with individual SNARC slopes. This finding also mirrors our recent observation that individual differences in SNARC effect strength cannot be explained by differential performance levels in a verbal working memory task (i.e. backwards digit recall; [94]). Whereas the “number access” hypothesis points to specific number treatment difficulties (which in turn weaken distracter inhibition), this last hypothesis points to a domain-general process. Of course, a theoretical framework combining above-mentioned factors is another possibility to provide a comprehensive explanation of the present findings.

In line with the hypotheses that inhibition processes play an important role in the strength of SNARC effects (see also [8], [24], [52]) math anxiety might also contribute to inter-individual differences in number-space associations. Math anxiety negatively influences arithmetical performance [95], [96] by affecting working memory performance [97]. It also decreases attentional control, which in turn diminishes inhibition capacities [98]. Whereas we tried to minimize the effects of math anxiety by administrating the simple parity judgment task first [95], [96], [99], we cannot definitely rule out that math anxiety might have influenced the results. Consequently it would be interesting to consider math anxiety as a possible variable impacting on the strength of the SNARC effect in future studies.

A final crucial consideration to be taken into account are possible inter-individual differences in the strategies used, as the use of different cognitive strategies could lead to differential SNARC effects in the three groups. In parity judgment tasks, the SNARC effect can for instance be associated with visuo-spatial as well as with verbal-spatial coding [11], [100]. Depending on their proficiency and training in mathematics, subjects could have employed different strategies to solve the task (see also [101]). Accordingly a training study by Delazer and colleagues [102] showed a shift of activation in the parietal lobe from the intraparietal sulcus (IPS) to the left angular gyrus (AG) after extensive training of complex multiplication problems. These findings suggest a shift from quantity-based processing to more automatic retrieval ([102]; see also the triple-code model of [103]). A differential study by Grabner and colleagues [104] showed that in healthy individuals, differing only in their mathematical competencies, higher achievers showed more left AG activation during single digit multiplication than their lower achieving peers. These findings were interpreted as high achievers relying more strongly on verbal strategies than low achievers ([104], see also [105], [106]). Similarly more trained subjects (i.e. ME) supposedly solve parity judgment employing more verbal strategies (similar to automatic fact retrieval) associated with left AG activation while less trained subjects (i.e. CON and MD) might rely more on quantity-based processes, thus activating more the IPS and the neighboring superior parietal regions critically involved in number-space interactions [107]–[110]. To date there are no studies investigating how the use of different strategies would modulate the strength of SNARC effects. To address these questions, future studies should explore how math proficiency levels influence dual task SNARC paradigms such as used by Van Dijck et al. [101] or Herrera and colleagues [111].

## Conclusion

The present study shows that the frequently reported inter-individual variance observed in the strength (and presence) of the SNARC effect is linked to mathematical proficiency. Participants that are more proficient in math have weaker SNARC effects in the classical parity judgment task. These findings could not be explained by general factors related to general processing speed, parity judgment reaction times or visuo-spatial working memory. We propose that they reflect individual differences concerning the access of numerical representations, as well as vulnerability to interference of irrelevant information.
